# Combination of Two Rapid Ophthalmic Test Kits for Improved Diagnosis in Cases of Severe Binocular Conjunctivitis

**DOI:** 10.3390/diagnostics10020109

**Published:** 2020-02-17

**Authors:** Asako Kodama, Fumitaka Kobayashi, Hao Yung Yang, Kazumi Fukagawa, Hiroyuki Yazu, Hiroshi Fujishima

**Affiliations:** 1 Department of Ophthalmology, Eiju General Hospital, Tokyo 110-8645, Japan; fumifumi_2323@hotmail.com; 2 Smile Eye Clinic, Kanagawa 227-0062, Japan; yang@hci.jp; 3 Ryogoku Eye Clinic, Tokyo 130-0062, Japan; fukakazu0706@gmail.com; 4 Department of Ophthalmology, Tsurumi University School of Dental Medicine, Kanagawa 230-0063, Japan; g.h.yazu@gmail.com (H.Y.); fujishima117@gmail.com (H.F.); 5 Department of Ophthalmology, International University of Health and Welfare Mita Hospital, Tokyo 108-8329, Japan; 6 Fujishima Eye Clinic, Niigata 949-6600, Japan

**Keywords:** conjunctivitis, allergic, adenovirus, diagnosis

## Abstract

Introduction: Diagnosis of conjunctivitis can be sometimes difficult, especially in cases of severe conjunctivitis and those involving both eyes. In this study, we performed commercial tests for adenovirus (Capilia Adeno Eye^®^) and total tear IgE (Allerwatch^®^) in a single visit in patients with bilateral conjunctivitis to examine if, and by how much, the combination of these two tests would improve the diagnostic accuracy of conjunctivitis. Methods: The study included sixty patients with relatively severe conjunctivitis in both eyes within a week of consulting our clinic and who had no previous treatment. Capilia Adeno Eye^®^ and Allerwatch^®^ tests were performed. Results: A significantly higher number of cases (55/60) were diagnosed when both tests were evaluated than with either test (Capilia Adeno Eye^®^ (12/60; *p* < 0.001) or Allerwatch^®^ (44/60; *p* < 0.005)) alone. The positivity rate of Allerwatch^®^ was significantly higher than that of Capilia Adeno Eye^®^ (*p* < 0.001). The diagnosis rate of atopic keratoconjunctivitis was 100% in patients with allergic conjunctivitis, but there was no significant difference in positivity compared with other types of allergic conjunctivitis. Conclusions: Testing patients with both Capilia Adeno Eye^®^ and Allerwatch^®^ improves the diagnostic accuracy for conjunctivitis and can diagnose more than 90% of cases. Detection of adenovirus antigen and IgE in tears, using these simple and rapid methods, will be useful for early diagnosis and prevention of adenoviral conjunctivitis.

## 1. Introduction

Conjunctivitis is typically infectious or allergic in origin. Infectious conjunctivitis is predominately viral or bacterial. In viral conjunctivitis, the source may be one of numerous viruses, including adenoviruses, herpes simplex virus, or picornavirus. Adenoviral keratoconjunctivitis is caused by many types of adenovirus with various clinical presentations. Though pharyngoconjunctival fever and follicular conjunctivitis are common, the most frequent is epidemic keratoconjunctivitis (EKC). EKC is characterized by photophobia, pain, lacrimation, discharge, and conjunctival injection and is most often caused by adenovirus type 4, 8, 19, or 37 [1]. Adenovirus is very resistant to desiccation and is transmitted by direct contact [2]. Therefore, prompt diagnosis is important. Several methods are available for the diagnosis of adenoviral keratoconjunctivitis (Table 1). Table 1 shows the advantages and disadvantages of each method. Adenoviral infection test kits are commercially available and useful for the diagnosis of EKC [3]. In one study, which consisted of 130 swabs from patients with conjunctivitis, two direct rapid tests to detect adenovirus antigen, immunochromatography (IC), and enzyme immunoassay (EIA) were compared with regard to sensitivity, specificity, and technical complexity; IC was quicker and easier than EIA and had higher specificity [4]. 

Allergic conjunctivitis can be categorized into seasonal (e.g., cedar pollen allergy), perennial (e.g., house-dust allergy), atopic keratoconjunctivitis (AKC), vernal keratoconjunctivitis (VKC), and giant papillary conjunctivitis. Seasonal and perennial are the most prevalent. VKC is a chronic ocular inflammatory process which can cause visual complications. VKC appears more frequently in young adults and children who have a history of asthma and seasonal allergy. Giant papillary conjunctivitis is a chronic immune response to long-term exposure to foreign bodies, such as contact lenses.

Allergic conjunctivitis and AKC are the most common ocular surface allergic diseases, affecting more than 20% of the population [5,6,7]. Recently, due to changes in living environment and other causes, the number of patients with allergic diseases, including atopic dermatitis and pollinosis, has been increasing. The most frequent complaints are itching, pain, lacrimation, discharge, and conjunctival injection in patients with relatively severe allergic conjunctivitis. Differential diagnosis of allergic conjunctivitis and AKC can be difficult. Several methods are available for the diagnosis of acute conjunctivitis (Table 1), all of which are time-consuming or require the use of a well-equipped laboratory; for example, options for serum testing require almost a week to obtain results and skin tests are quite limited for ophthalmologists. However, a new commercial kit (Allerwatch^®^, Hitachi Chemical Co., Ltd., Tokyo, Japan, and Wakamoto Pharmaceutical Co., Ltd., Tokyo, Japan) has been developed and released recently that provides a rapid immunoassay for measuring total tear IgE. The measurements of allergen-specific serum IgE and total tear IgE are considered useful for diagnosis [8,9]. Several research groups have also reported that measuring total tear IgE by IC can be a useful tool for diagnosis in cases of doubt [10].

Adenoviral keratoconjunctivitis and allergic conjunctivitis are the common causes of bilateral conjunctivitis [11]. The accurate diagnosis of a patient with conjunctivitis starts with a careful medical history assessment, with a systematic ophthalmic examination. To differentiate between the two diseases and come to a firm diagnosis can sometimes be difficult when historical features and examination findings are less clear [6,12]. In clinical practice, the more likely cause of the disease is tested first. However, if tested negative, it may result in diagnostic delays. We expect more accurate results when performing two tests at the same time. Nevertheless, no studies have investigated the diagnostic usefulness of performing two tests in a single visit in patients with bilateral conjunctivitis. For this study, we recruited patients with moderate-to-severe bilateral conjunctivitis that was difficult to diagnose due to unclear medical history and examination findings. We performed two rapid tests for adenovirus and total tear IgE in a single visit and examined if, and by how much, the combination of these two tests would improve diagnostic accuracy. In this paper, we describe the relevant instrumentation and measurement techniques. 

## 2. Patients and Methods

### 2.1. Study Design

**Figure 1:**
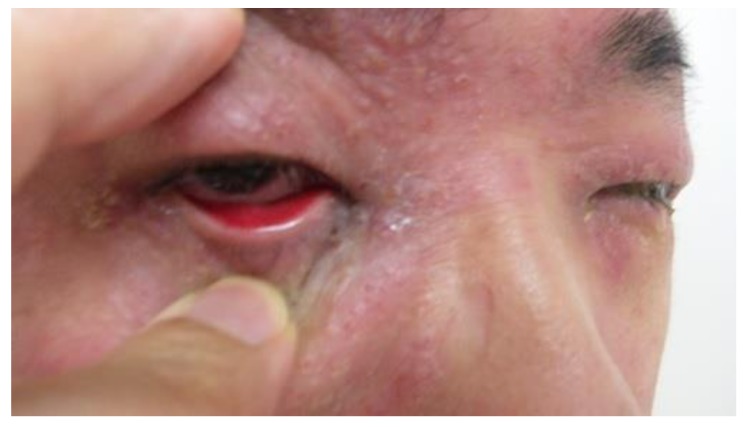
Patients with binocular conjunctivitis. Conjunctival injection and swelling of the eyelids occur in both eyes.

Sixty patients (39 men and 21 women; age range: 21 to 58 years; mean ± SD age, 29.5 ± 24.2 years) with binocular conjunctivitis and debilitating ocular symptoms, including itching, tearing, photophobia, or discharge, were enrolled in this study (Figure 1). Chemosis, conjunctival injection, and swelling of the eyelids commonly occur in association with these symptoms. Conjunctivitis was diagnosed by a single ophthalmologist (H.F.), according to published guidelines for the diagnosis and treatment of conjunctivitis [13]. In brief, the diagnosis was based on a history of symptoms (e.g., ocular itching, pain, discharge, and tearing) and slit lamp examination findings (e.g., conjunctival injection, follicles, and papillae). Patients were enrolled if they had moderate-to-severe bilateral conjunctivitis that was difficult to diagnose due to unclear medical history and examination findings. We performed two rapid tests for adenovirus and total tear IgE in a single visit and evaluated the results. Any other types of conjunctivitis, such as those caused by foreign bodies or Stevens–Johnson syndrome were excluded. Patients with proliferative AKC were also excluded. None of the patients wore contact lenses or had been treated with a topical or systemic drug in the preceding month. This study was a prospective, non-randomized, consecutive case study conducted at the Fujishima Eye Clinic in Niigata prefecture, Japan. The study was performed in accordance with the Helsinki Declaration of 1975 and its later amendments. Approval was obtained from the Institutional Review Board at International University of Health and Welfare Mita Hospital (Tokyo, Japan; IRB No. H21-009, September 1, 2009). The clinical trial registration ID number is UMIN 000035825. Informed consent was provided by each subject.

Rapid diagnosis of adenoviral conjunctivitis on conjunctival swabs with 15 min IC for adenovirus detection was used for the differential diagnosis of EKC (Capilia Adeno Eye^®^; Wakamoto Pharmaceutical Co., Tokyo, Japan). This test has demonstrated high sensitivity and specificity [14]. Rapid diagnosis of allergic conjunctivitis with a new commercial test for total tear IgE, based on IC (Allerwatch^®^; Wakamoto Pharmaceutical Co., Tokyo, Japan), was performed for the differential diagnosis of allergic conjunctivitis. High sensitivity and specificity of this test have been reported [15]. Samples were collected from the most severely affected eye.

### 2.2. Determination of Adenovirus from Conjunctival Sac and IgE in Tears

#### 2.2.1. Capilia Adeno Eye^®^

**Figure 2:**
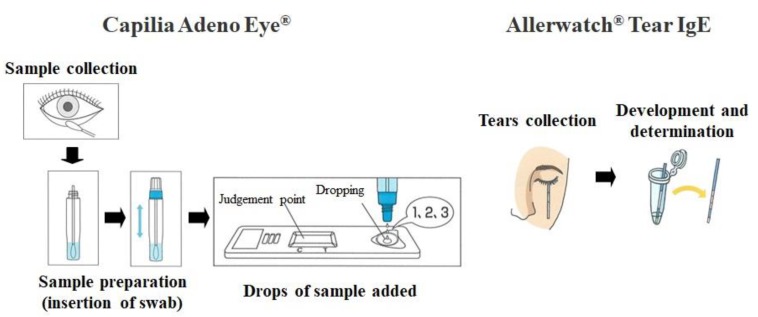
Test procedures for Capilia Adeno Eye^®^ and Allerwatch^®^. Capilia Adeno Eye^®^: The corneal conjunctiva is swabbed, the swab is immersed in the extracting solvent, and drops of the solvent are added to a plate for determination of the presence of adenovirus, within approximately 15 min; Allerwatch^®^ Tear IgE: After collecting tears by inserting the test paper strip into the conjunctival sac behind the lower eyelid, the test paper is immersed in the developing solvent within approximately 10 min. The test paper strip is removed, and the result is evaluated.

Capilia Adeno Eye^®^ (Figure 2) is a quick, easy-to-perform diagnostic kit for adenovirus that uses IC. The corneal conjunctiva is swabbed, the swab is immersed in the extracting solvent, and drops of the solvent are added to a plate for determination of the presence of adenovirus within approximately 15 min. The plate contains reagents necessary for detection of adenovirus, and when adenovirus is present in the extracting solvent, a red line develops in the specified position. 

#### 2.2.2. Allerwatch^®^ Tear IgE 

Allerwatch^®^ Tear IgE (Figure 2) is the first quick, easy-to-perform diagnostic kit in Japan for allergic conjunctivitis that uses IC, which assesses total IgE in tears. After collecting tears by inserting the test paper strip into the conjunctival sac behind the lower eyelid, the test paper is immersed in the developing solvent within approximately 10 min. The test paper contains reagents necessary for measurement of total IgE in the tears, and when IgE is present, a red line develops in the specified position. The intensity of the red color is dependent on the content of total IgE. No line appears at normal IgE levels (<2.0 KU/L), as instructed by the manufacturer’s manual. The test results are categorized into three grades: Grade 0 = no detectable IgE (no line), Grade 1 = a lower total IgE level (test line weaker than the control), and Grade 2 = a higher total IgE level (test line similar or stronger than the control). The results in this study were determined by a participating doctor. 

### 2.3. Statistical Analysis

Data were expressed as absolute values. Commercially available software (StatView, version 5.0; SAS Institute Japan, Tokyo, Japan) was used for all statistical analyses. The Fisher’s exact test was used for comparisons, and *p* values < 0.05 were considered statistically significant.

## 3. Results

**Figure 3:**
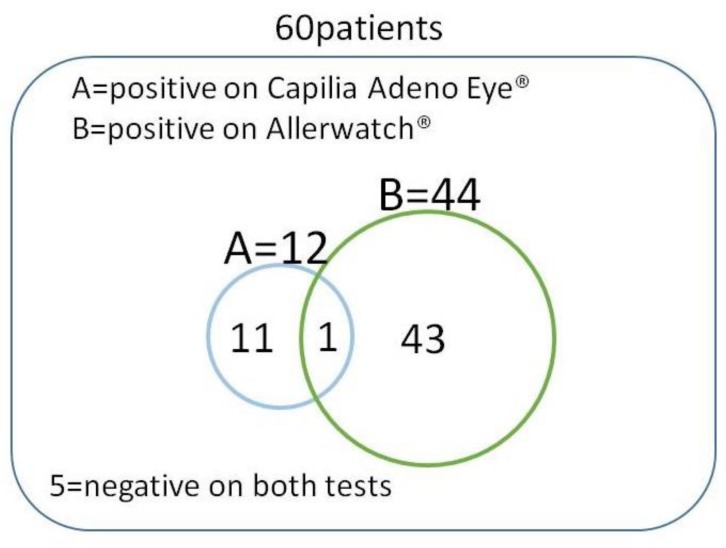
Positive distribution of Capilia Adeno Eye^®^ and Allerwatch^®^. Of the 60 patients enrolled, 55 (91.7%) had positive results on at least one test, and one patient tested positive on both tests.

**Figure 4:**
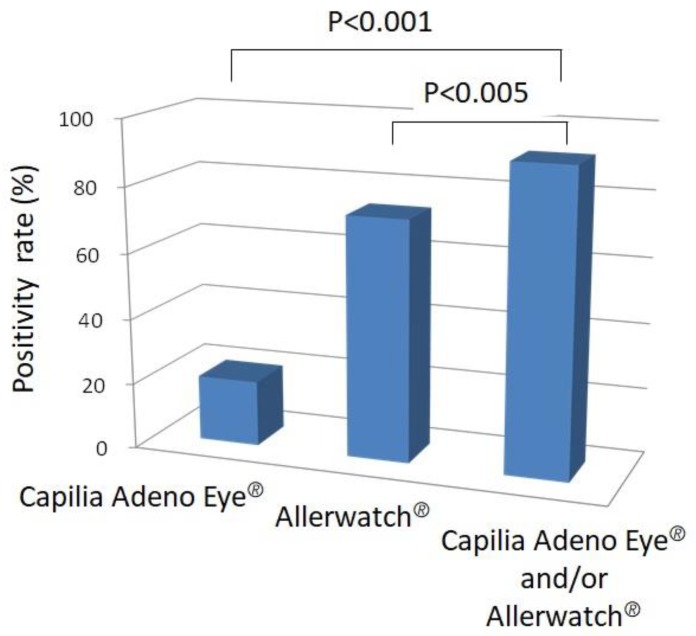
Positivity rate with Capilia Adeno Eye^®^ alone, Allerwatch^®^ alone, and Capilia Adeno Eye and/or Allerwatch^®^. A significantly higher number of cases were diagnosed when both tests were evaluated than with either Capilia Adeno Eye^®^ (*p* < 0.001) or Allerwatch^®^ (*p* < 0.005) alone.

**Figure 5:**
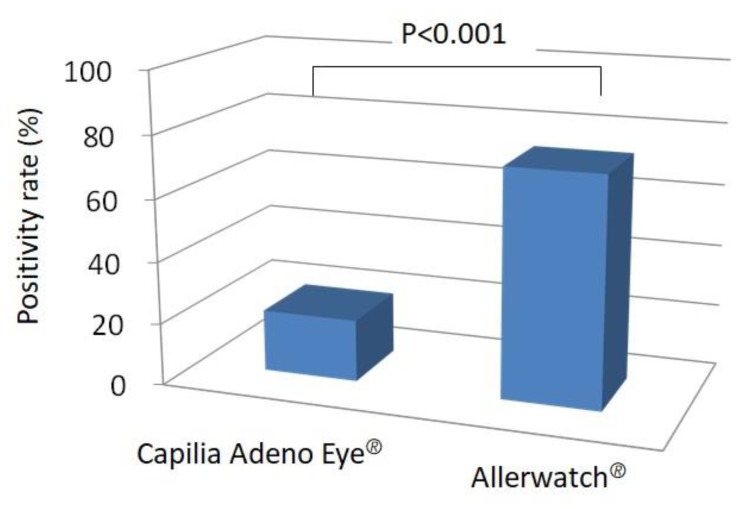
Positivity rate with Capilia Adeno Eye^®^ and Allerwatch^®^. The positivity rate with Allerwatch^®^ was significantly higher than with Capilia Adeno Eye^®^ (*p* < 0.001).

Of the 60 patients enrolled, 12 tested positive for Capilia Adeno Eye^®^ and 44 for Allerwatch^®^. Among the 60 patients, 55 had positive results on at least one test, and one patient tested positive on both tests (Table 2 and Figure 3). A significantly higher number of cases (55/60, 91.7%) were diagnosed when both tests were evaluated than with either Capilia Adeno Eye^®^ (12/60, 20%) (*p* < 0.001) or Allerwatch^®^ (44/60, 73.3%) (*p* < 0.005, Table 2 and Figure 4) alone. Thirty-eight patients (38/60, 63.3%) tested highly positive, and six (6/60, 10.0%) tested slightly positive with Allerwatch^®^ (Table 2). The positivity rate with Allerwatch^®^ was significantly higher than with Capilia Adeno Eye^®^ (*p* < 0.001, Table 2 and Figure 5). In patients with allergic conjunctivitis, all seven patients with AKC were diagnosed, but there was no significant difference in positivity in other allergic conjunctivitis cases. Five of 60 (8.3%) patients were negative on both tests. One patient tested slightly positive with Capilia Adeno Eye^®^ and also Allerwatch^®^. No side effects were reported among patients who had two tests performed. Patients were treated with suitable eye drops (e.g., antihistamine eye drop and steroid eye drop for allergic conjunctivitis) and healed without any complications.

## 4. Discussion

In this study, we examined two kits for the clinical diagnosis of relatively severe bilateral conjunctivitis. Using both tests together, 91.7% of conjunctivitis cases were diagnosed. This percentage was significantly higher than those obtained by using only one test. Of course, in cases of bilateral conjunctivitis, viral infection and allergy are known to be the common causes [11]; this experiment showed that using two tests provides more reliable results than either test alone. There are those cases where we make a tentative diagnosis being unsure, whether it is initial allergic conjunctivitis with small papillae or viral conjunctivitis with small follicles in the conjunctiva. In clinics, there are cases when these two entities may present in the same patient as well. Thus, these testing kits may help the clinicians differentiate these two etiologies in challenging cases.

The positivity rate of the tear IgE test was significantly higher than that of the adenovirus test. We think this result is due to the disease prevalence rate. Patients with allergic conjunctivitis are more prevalent than patients with EKC.

The diagnosis rate for AKC was 100% in seven patients with atopic dermatitis, using Allerwatch^®^. AKC is a chronic atopic disease of the cornea and conjunctiva. AKC is associated with atopic dermatitis and occurs in both adults and children. Owing to its chronicity and ability to induce loss of vision due to various corneal complications, AKC is the most debilitating of the allergic conjunctivitis. The immunopathogenic mechanisms of AKC are complex; its signs and symptoms can be related to several effector cells, such as T cells, mast cells, eosinophils, and, more recently, conjunctival epithelial cells. AKC is frequently refractory to treatment [16,17,18]. Thus, AKC is thought to be a Th2- and IgE-related disease. IgE production in tears might be greater than in other types of perennial allergic conjunctivitis [19]. 

Even though AKC was diagnosed in 100% of the cases, there was no significant difference in positivity for other types of allergic conjunctivitis. Seasonal allergic conjunctivitis is the most prevalent of the allergic disorders that afflict the ocular surface. In Japan, cedar pollen allergy is the most important allergic disease, causing severe itching in afflicted patients [8,20]. This characteristic might be one reason for the lack of a significant difference compared with diagnosis of AKC.

The gold standard for the positive diagnosis of allergic conjunctivitis is the presence of eosinophils on cytologic examination. However, this type of test can be difficult. Allerwatch^®^ is a quick diagnostic test kit that uses IC for allergic conjunctivitis; it is the first in Japan to allow detection of IgE in tears of the patients (Figure 2) [21]. Allerwatch^®^ is a minimally invasive kit for rapid diagnosis of allergic conjunctivitis, directly reflecting local eye allergy and requiring no special devices. It has high specificity [15], and, therefore, a positive result with Allerwatch^®^ may be sufficient for diagnosis of allergic conjunctival disease without cytologic examination. Capilia Adeno Eye^®^ is another quick, easy-to-perform diagnostic kit. Unlike other test kits, the sample can be prepared by encapsulating the collected swab in a container of the extracting solvent, which makes the operation easier. Moreover, the risk of nosocomial infection caused by aerosols is expected to be decreased because the kit can be discarded with the swab encapsulated in the container. These two kits could be used in otolaryngology and pediatrics by using per-nasal swabs.

The single patient who tested positive on both tests is an unusual case. We think this patient was suffering from AKC and EKC at the same time. In such a case, the initial treatment must be for EKC, followed by treatment for AKC [2,22]. As for the five patients who tested negative on both tests, we think the results could be due to technical error, limits of these examinations, or other infectious diseases. These patients had bilateral disease, which makes foreign bodies or bacterial infections unlikely.

## 5. Conclusions

As discussed above, the two test kits are useful for the clinical diagnosis of relatively severe bilateral conjunctivitis that was difficult to diagnose due to unclear medical history and examination findings. However, Allerwatch^®^ is not an antigen-specific test, and Capilia Adeno Eye^®^ responds selectively to only adenovirus. Because avoidance of the offending antigen is the primary behavioral modification for all types of allergic conjunctivitis, Allerwatch^®^ is expected to be improved, and it will be easier to detect the specific allergen. We also expect the development of new testing equipment, which detects other viruses, as well as adenovirus. 

## Figures and Tables

**Table 1:** Advantages and disadvantages of the methods for the diagnosis of conjunctivitis.

**Adenoviral Keratoconjunctivitis**		
	**Advantages**	**Disadvantages**
Cytologic examination	rapid	quite limited for the clinic
(mononuclear cells)		auxiliary diagnosis
Genetic testing	rapid	require the well-equipped laboratory
(e.g., nested-PCR, real-time PCR)	high sensitivity	
Test kit (e.g., Capilia Adeno Eye^®^)	rapid	limited accuracy
(virus antigen)	easy-to-perform	
**Allergic Conjunctivitis**		
	**Advantages**	**Disadvantages**
Cytologic examination	rapid	quite limited for the clinic
(eosinophils)	confirmed diagnosis	
Serum testing	confirmed diagnosis	time-consuming
(allergen-specific serum IgE)		require the well-equipped laboratory
Test kit (e.g., Allerwatch^®^)	rapid	limited accuracy
(total tear IgE)	easy-to-perform	

PCR, polymerase chain reaction.

**Table 2:** Results of Capilia Adeno Eye^®^ and Allerwatch^®^.

	Capilia Adeno Eye^®^	Allerwatch^®^	Capilia Adeno Eye^®^ and/or Allerwatch^®^
Positive	12	Grade2, 38	55
		Grade1, 6	
Negative	48	Grade0, 16	5
Positivity Rate	12/60 (20%)	44/60 (73.3%)	55/60 (91.7%)

## Abbreviations

EKC	Epidemic keratoconjunctivitis
IC	Immunochromatography
EIA	Enzyme immunoassay
AKC	Atopic keratoconjunctivitis
VKC	vernal keratoconjunctivitis
PCR	Polymerase chain reaction
